# Breaking the Cycle of Polypharmacy: A Case Report of Renal Denervation in Resistant Hypertension

**DOI:** 10.3390/jcm15155838

**Published:** 2026-07-26

**Authors:** Maria Szwarkowska, Tymoteusz Petela, Aleksander Zeliaś, Tomasz Skowerski, Tomasz Tokarek

**Affiliations:** 1 Centre for Innovative Medical Education, Jagiellonian University Medical College, Medyczna 7, 30-688 Kraków, Poland; maria.szwarkowska@student.uj.edu.pl; 2 Students’ Scientific Group of Centre for Innovative Medical Education, Jagiellonian University Medical College, 30-688 Kraków, Poland; 3 Faculty of Medical Sciences in Zabrze, Medical University of Silesia in Katowice, Traugutta 2, 41-800 Zabrze, Poland; s88123@365.sum.edu.pl; 4 Faculty of Medicine and Health Science, University of Applied Science in Nowy Sącz, 33-300 Nowy Sącz, Poland; aa.zelias@gmail.com; 5 2nd Clinical Department of Cardiology, Faculty of Medicine and Health Science, University of Applied Science in Nowy Sącz, 33-300 Nowy Sącz, Poland; 6 Department of Cardiology, School of Health Sciences in Katowice, Medical University of Silesia, 40-055 Katowice, Poland; 7 Centre for Invasive Cardiology, Electrotherapy and Angiology, Kilińskiego 68, 33-300 Nowy Sącz, Poland

**Keywords:** hypertension, medication deescalation, blood pressure monitoring, nephron-sparing surgery, cardio-nephrology

## Abstract

**Background**: Resistant hypertension poses a significant therapeutic challenge, often leading to severe polypharmacy. Renal denervation (RDN) has re-emerged as a valuable adjunctive intervention for blood pressure control. **Case Presentation**: We report the case of a 64-year-old man (body mass index [BMI] 34 kg/m^2^) with long-standing resistant hypertension (RH), after previous percutaneous coronary intervention (PCI) to the left anterior descending artery, heart failure with preserved ejection fraction (HFpEF), and prior nephron-sparing surgery for clear cell renal carcinoma. Despite treatment with an extensive antihypertensive regimen encompassing nine pharmacological classes including diuretic therapy (angiotensin-converting enzyme inhibitor; calcium channel blocker, thiazide diuretic, β-blocker, α1-blocker, central α2-agonist, mineralocorticoid receptor antagonist, loop diuretic, long-acting nitrates), blood pressure remained severely uncontrolled on both home and office measurements. Persistent hypertension was accompanied by exertional dyspnoea and episodes of exertional chest discomfort. Following comprehensive evaluation and exclusion of secondary causes of hypertension, the patient underwent catheter-based renal denervation using the SymplicitySpyral™ (Medtronic) multi-electrode radiofrequency system. The procedure was associated with substantial and sustained improvement in blood pressure control, with mean 24 h ambulatory blood pressure measurements decreasing to 130/80 mmHg at six-month follow-up. Importantly, successful blood pressure reduction enabled major simplification of pharmacotherapy, including complete discontinuation of clonidine, loop diuretic therapy, and long-acting nitrates, together with marked dose reduction in doxazosin. **Conclusions**: This case illustrates the potential clinical utility of renal denervation in carefully selected patients with true resistant hypertension and pronounced sympathetic overactivity. Beyond achieving satisfactory blood pressure control, RDN may facilitate meaningful reduction in medication burden, potentially improving treatment adherence, quality of life, and long-term cardiovascular risk. Written informed consent was obtained from the patient for both the procedure and the publication of this case report.

## 1. Introduction

Hypertension remains one of the leading modifiable contributors to cardiovascular morbidity and mortality worldwide [1,2,3]. Resistant hypertension (RH) represents a particularly challenging clinical phenotype. It is currently defined as blood pressure remaining above guideline-recommended targets despite treatment with at least three antihypertensive agents of complementary mechanisms, including a diuretic, prescribed at maximally tolerated doses [1,2,3,4,5]. Patients whose blood pressure is controlled only with four or more medications are also considered to have RH [1,2,3]. Contemporary epidemiological data suggest that RH affects 10–20% of treated hypertensive patients [1,3].

Compared with patients achieving adequate control using conventional pharmacotherapy, individuals with RH exhibit substantially increased rates of stroke, chronic kidney disease progression, coronary artery disease, atrial fibrillation, and heart failure, particularly HFpEF [1,3].

The pathophysiology of RH is multifactorial and incompletely understood; however, persistent sympathetic nervous system (SNS) activation plays an important mechanistic role [1,4]. Excessive renal sympathetic efferent signalling promotes renin release, sodium retention, renal vasoconstriction, and volume expansion, whereas afferent renal nerve activity augments central sympathetic outflow, thereby perpetuating systemic hypertension [5]. Concomitant activation of the renin–angiotensin–aldosterone system (RAAS) further contributes to vascular remodelling, endothelial dysfunction, and target-organ injury [6].

Before establishing the diagnosis of true RH, pseudo-resistance must be excluded [1,7]. This includes inadequate blood pressure measurement technique, medication non-adherence, white-coat hypertension, and suboptimal therapeutic regimens [1,7]. Current guidelines strongly recommend confirmation using ambulatory blood pressure monitoring (ABPM) or validated home blood pressure measurements [1,4]. A systematic evaluation for secondary causes of hypertension is equally essential. It should include screening for primary aldosteronism, renal artery stenosis, obstructive sleep apnoea, pheochromocytoma, Cushing syndrome, and chronic kidney disease [1,2].

In appropriately selected patients with confirmed RH despite optimal medical therapy and lifestyle intervention, catheter-based renal denervation (RDN) has emerged as a potential adjunctive therapeutic strategy [1,4,8,9]. Recent sham-controlled trials using second-generation multi-electrode systems have demonstrated sustained reductions in blood pressure in both clinic-based measurements and 24 h ambulatory blood pressure monitoring (ABPM), with an acceptable safety profile [9].

However, clinical data regarding the safety and efficacy of RDN remain extremely limited in patients with a severely reduced renal mass and an exceptionally high pill burden. Patients who have undergone prior partial nephrectomy for renal cell carcinoma present a unique pathophysiological and technical challenge; tissue scarring and structural alterations within the remaining renal parenchyma may amplify pathological afferent sympathetic signalling, yet data on treating such individuals are scarce.

We present a case of severe RH in a patient with a history of partial nephrectomy and a complex cardiovascular profile. This case demonstrates the utility of radiofrequency RDN using a multi-electrode system to break the cycle of extreme polypharmacy, resulting not only in substantial blood pressure control but also in a major de-escalation of complex antihypertensive therapy.

## 2. Case Presentation

### 2.1. Patient Information and Clinical Findings

A 64-year-old man (height 187 cm, weight 120 kg, BMI 34 kg/m^2^) with long-standing RH was referred for further evaluation after persistently uncontrolled blood pressure despite progressive intensification of pharmacotherapy over six months. The patient demonstrated excellent adherence to both lifestyle and pharmacological recommendations, including intentional weight reduction of approximately 14 kg. Nevertheless, home blood pressure measurements consistently ranged between 160–180/90–110 mmHg.

His medical history was significant for:

- coronary artery disease with prior PCI to the left anterior descending artery (LAD) (2016);
- HFpEF;
- partial nephrectomy for clear cell renal cell carcinoma performed one year earlier;
- type 2 diabetes mellitus;
- dyslipidemia;
- obstructive sleep apnoea treated with continuous positive airway pressure (CPAP);
- chronic gastritis;
- stage I hypertensive angiopathy.

Metabolic control had improved substantially during optimisation of antihypertensive therapy, with glycated haemoglobin decreasing from 8.1% to 7.0%. Low-density lipoprotein cholesterol was well controlled at 1.4 mmol/L.

At presentation, the patient reported exertional dyspnoea and episodic retrosternal chest tightness radiating toward the neck. Office blood pressure measurements reached 230/130 mmHg. Heart rate was 80 beats/m. Physical examination revealed no abnormal findings.

### 2.2. Timeline

**Figure 1:**
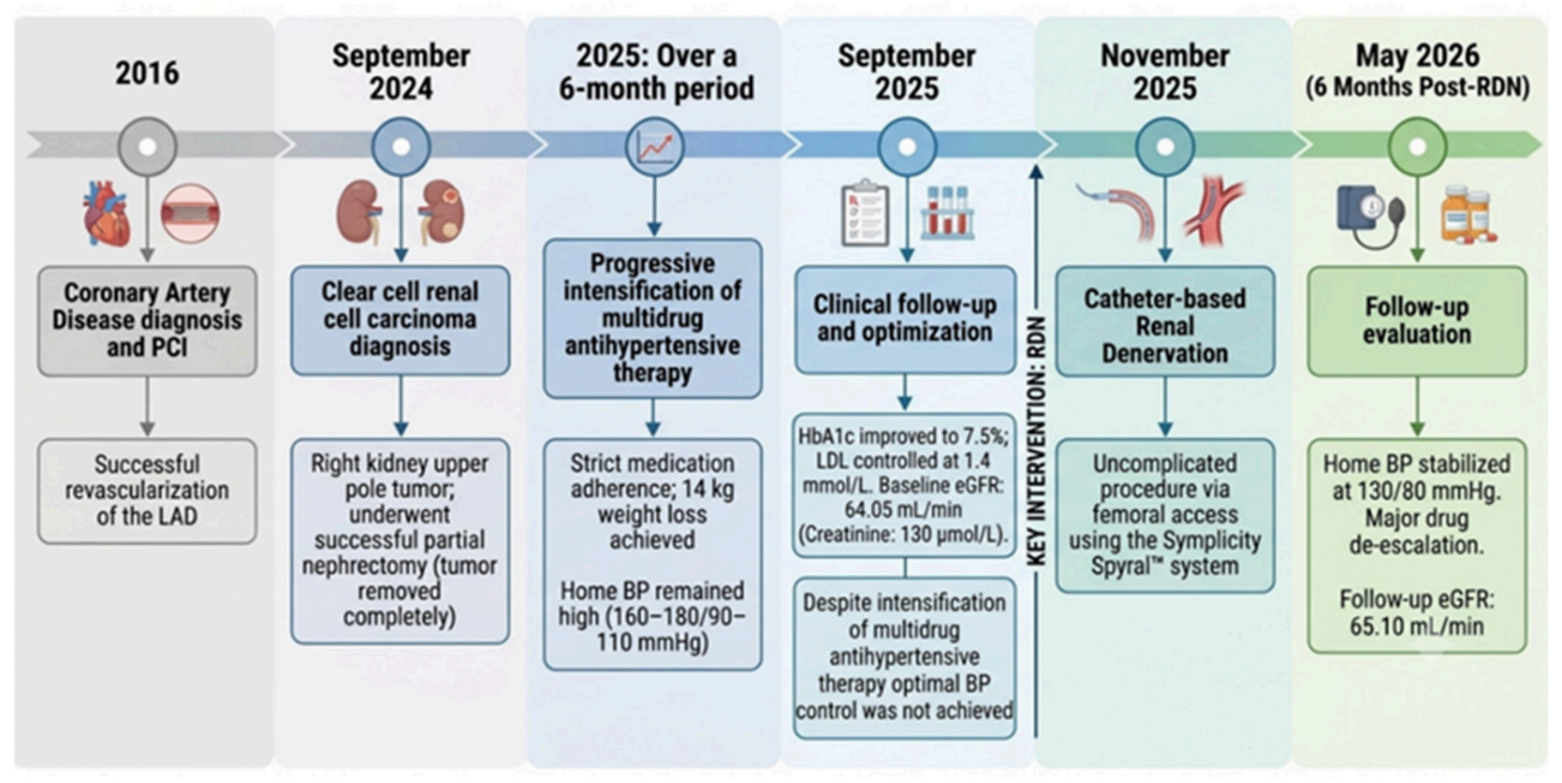
A schematic diagram illustrating the chronological timeline of the case.

The chronology of the main clinical events, interventions, and follow-ups is summarised in Figure 1.

### 2.3. Diagnostic Assessment

A baseline laboratory test confirmed mildly decreased kidney function with a baseline serum creatinine level of 130 μmol/L and an estimated glomerular filtration rate (eGFR) of 64.05 mL/min/1.73 m^2^, which was expected given his history of a prior partial nephrectomy. The remaining laboratory results were within normal limits. Secondary causes of hypertension (including primary aldosteronism, renal artery stenosis, and other endocrine disorders) were systematically excluded. Medication compliance was strongly confirmed via detailed longitudinal clinical interviews.

Transthoracic echocardiography demonstrated mild left atrial enlargement and left ventricular dilatation (left ventricular end-diastolic diameter—6.3 cm), mild concentric left ventricular hypertrophy with interventricular septal thickness of 1.3 cm and preserved left ventricular systolic function (LVEF 60%). After gradual blood pressure decrease, an exercise treadmill ECG stress testing was negative for inducible myocardial ischaemia; however, a markedly exaggerated hypertensive response was observed, with peak blood pressure reaching 220/110 mmHg.

At the time of evaluation, the patient was receiving extensive antihypertensive therapy consisting of 9 distinct pharmacological agents (totalling 13 pills daily):

- ramipril 10 mg once daily (OD);
- amlodipine 10 mg OD;
- hydrochlorothiazide 25 mg OD;
- bisoprolol 10 mg od;
- doxazosin 8 mg twice daily;
- clonidine 150 μg three times daily;
- spironolactone 25 mg OD;
- torasemide 20 mg OD;
- isosorbide mononitrate 80 mg OD.

### 2.4. Therapeutic Intervention

**Figure 2:**
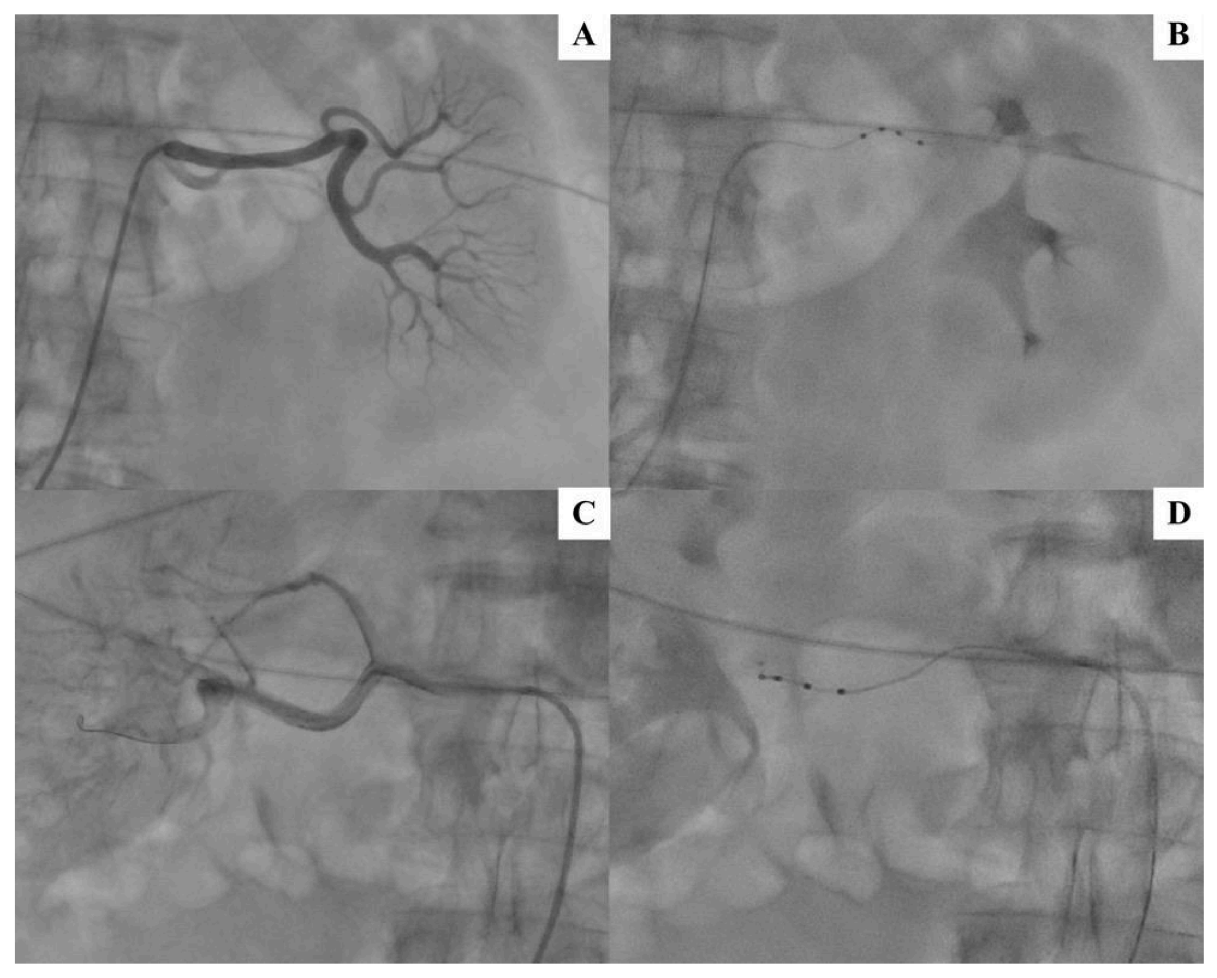
Renal angiography and catheter-based renal denervation. Preprocedural angiography confirmed suitable anatomy of the right and left renal arteries without significant stenosis. Images (**A**,**C**) show selective angiography of the left and right renal arteries, respectively. Images (**B**,**D**) demonstrate positioning of the Symplicity Spyral™ catheter during radiofrequency ablation in the left and right renal arteries, respectively.

**Figure 3:**
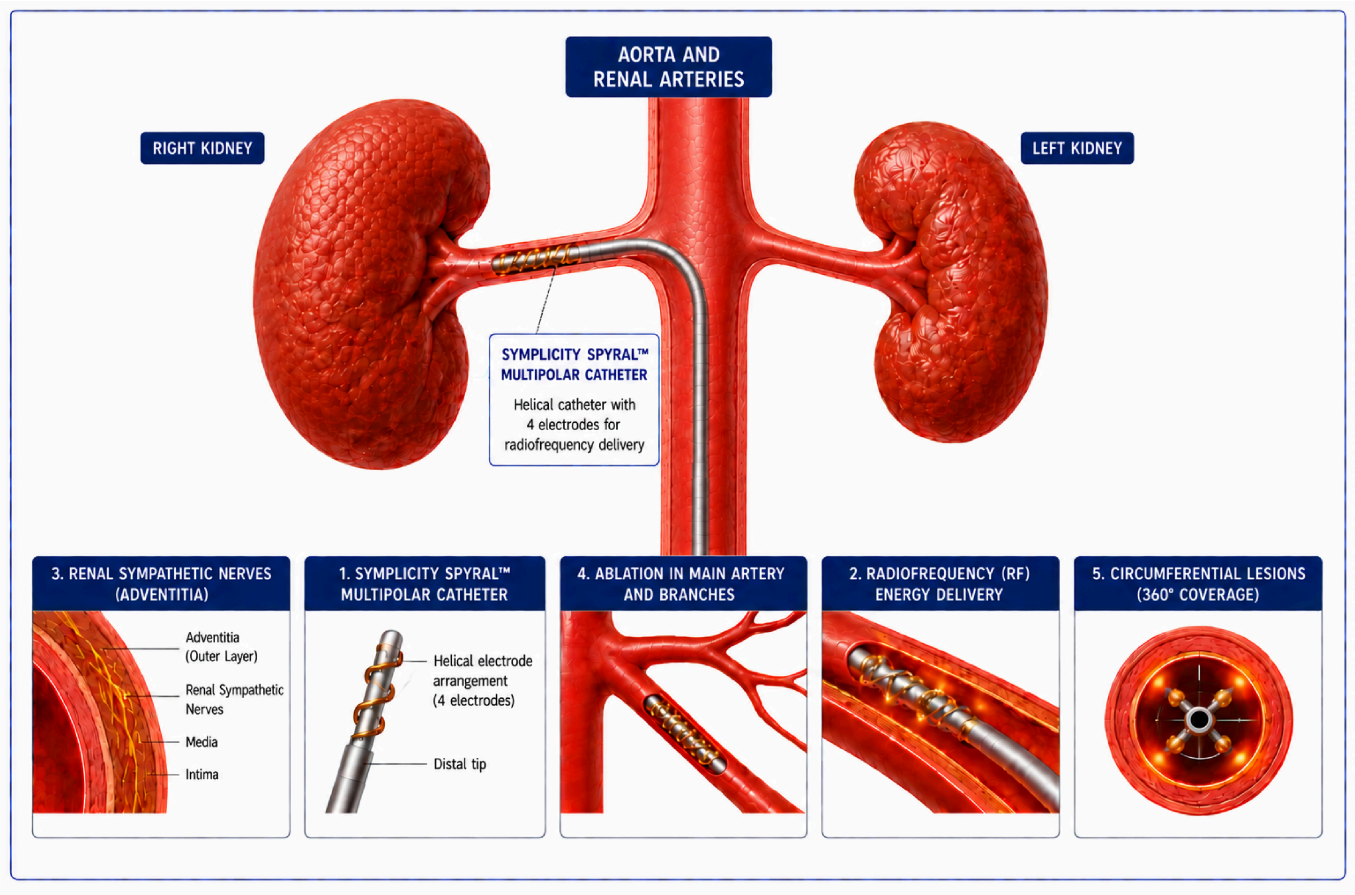
Schematic illustration of catheter-based renal denervation (RDN) using the Symplicity Spyral™ multi-electrode radiofrequency catheter system (Medtronic).

Given persistent symptomatic severe hypertension despite maximally tolerated multidrug therapy, the patient was referred for catheter-based renal denervation. Antihypertensive therapy remained stable for several weeks before the procedure. Preprocedural angiography confirmed suitable bilateral renal artery anatomy without significant stenosis—Figure 2. The procedure was performed via femoral arterial access using the 4F SymplicitySpyral™ multi-electrode radiofrequency catheter system (Medtronic)—Figure 1; a scheme of the procedure is presented in Figure 3. A total of 12 radiofrequency applications were delivered to the left renal artery and 23 to the right renal artery, including branch vessel ablations were anatomically feasible. During the procedure, a total of 100 mL of Ultravist contrast medium was administered. Total fluoroscopy time was 15 min, with a cumulative radiation dose of 363 mGy. Intraoperative pharmacology included 10,000 IU of heparin and 0.5 mg of remifentanil for sedation and analgesia. Vascular closure was achieved using an Angio-Seal™ 6F device. The procedure was uncomplicated.

### 2.5. Follow-Up and Outcomes

**Figure 4:**
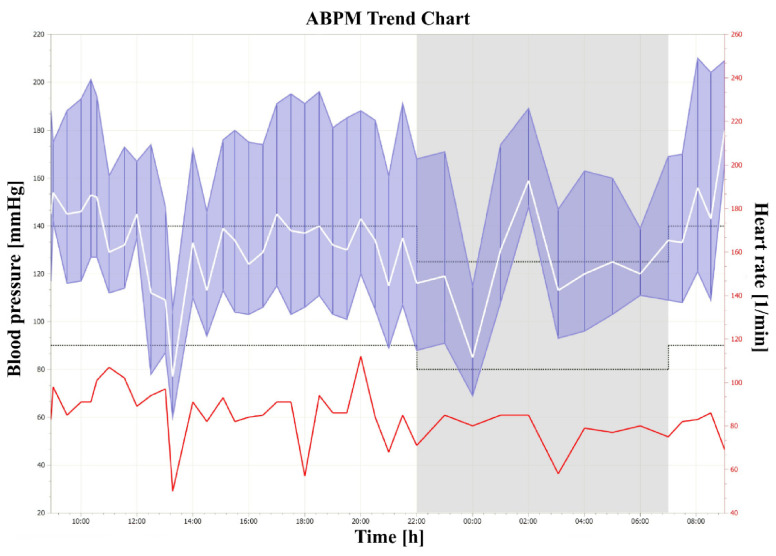
24 h ambulatory blood pressure monitoring before first sympathetic renal denervation. Black and gray dots represent normal range for 24 h ambulatory blood pressure monitoring. The red line represents heart rate.

**Figure 5:**
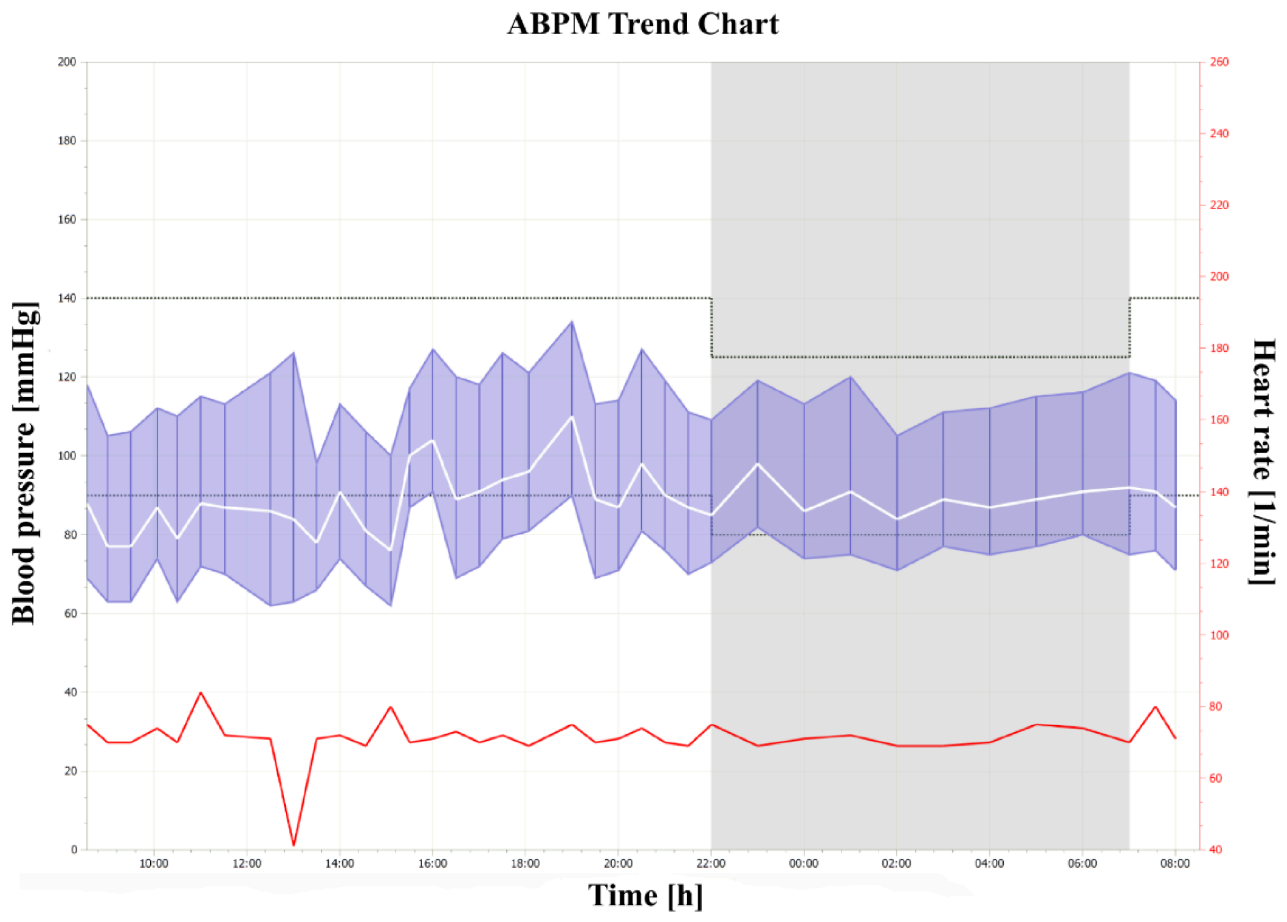
24 h ambulatory blood pressure monitoring after first sympathetic renal denervation, 6 months after the procedure Black and gray dots represent normal range for 24 h ambulatory blood pressure monitoring. The red line represents heart rate.

Follow-up ambulatory blood pressure monitoring (ABPM) performed six months after the intervention demonstrated a profound, clinically significant improvement in blood pressure control, with mean 24 h values successfully stabilised at approximately 130/80 mmHg (Figure 4 and Figure 5). Notably, the pathological nocturnal blood pressure variability, the blunted nocturnal dipping status, and the exaggerated morning blood pressure surge were markedly attenuated.

Crucially, given the patient’s history of a partial nephrectomy, post-procedural renal function was meticulously monitored. Repeated laboratory evaluations confirmed that the procedure caused no deterioration in renal filtration function; serum creatinine remained completely stable at 128 umol/L with a corresponding eGFR of 65.10 mL/min/1.73 m^2^ at the 6-month evaluation, providing objective evidence of procedural safety.

**Figure 6:**
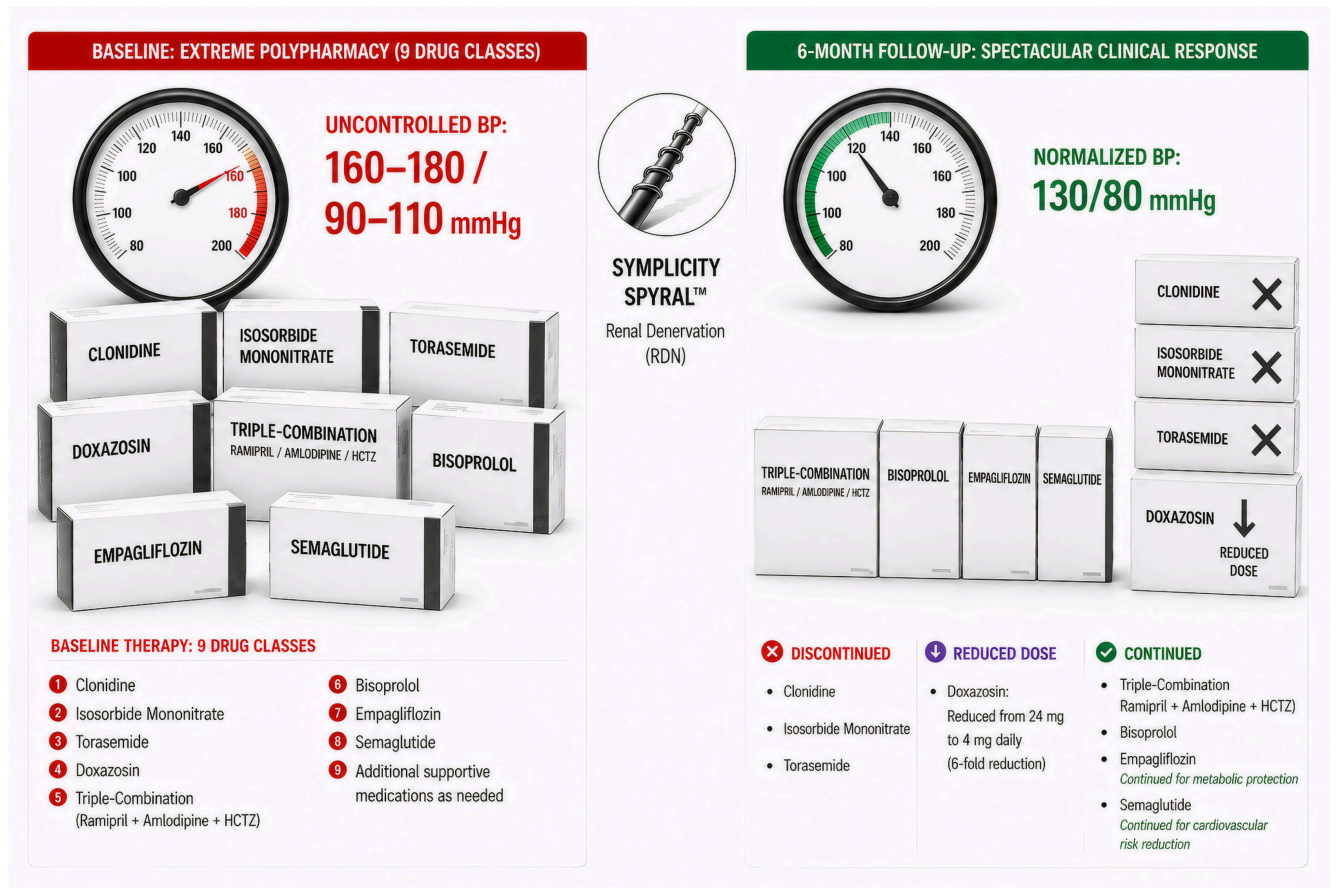
Schematic comparison of pharmacotherapy before and after renal denervation (RDN).

This favourable hemodynamic response enabled a dramatic simplification of his complex regimen and successfully broke the cycle of extreme polypharmacy. Four distinct classes of antihypertensive medications—clonidine (centrally acting agent), torasemide (loop diuretic), spironolactone (mineralocorticoid receptor antagonist), and long-acting isosorbide mononitrate (nitrate)—were discontinued entirely. Additionally, the daily dose of the alpha-1 blocker doxazosin was reduced by 75% (from 16 mg down to a single 4 mg dose) (Figure 6).

Concomitant non-antihypertensive treatments, including acetylsalicylic acid (75 mg OD), rivaroxaban (2.5 mg BID), and rosuvastatin with ezetimibe (40+10 mg OD), were maintained. Metformin (long-acting form—2g OD), empagliflozin (10 mg OD), and semaglutide (titrated up to 1 mg subcutaneously once weekly) were securely maintained owing to their established, independent long-term cardiometabolic benefits.

### 2.6. Patient Perspective

The patient reported a significant, highly notable improvement in his overall quality of life and functional capacity. He noted the complete resolution of episodic chest tightness and exertional dyspnoea. He expressed great satisfaction with the clinical outcomes, emphasising that the reduction in his daily medication burden—from a complex, multi-dose regimen to a simplified routine—had significantly improved his mental wellbeing, restored his vitality, and eliminated the constant anxiety associated with uncontrolled, sudden spikes in blood pressure.

## 3. Discussion

This case illustrates several clinically important aspects of contemporary RH management. First, it highlights the substantial burden of polypharmacy frequently encountered in patients with true RH [10]. Despite the availability of multiple antihypertensive drug classes, in some patients’ arterial pressure remains inadequately controlled even with complex multidrug regimens, exposing them to increased risk of adverse drug reactions, impaired adherence, and diminished quality of life [4,10,11]. In our patient, the initial treatment regimen consisted of nine different pharmacological agents, which resulted in an extremely high medication burden (13 pills per day) and likely impaired adherence and therapeutic success before the intervention.

Second, this case underscores the importance of sympathetic nervous system hyperactivity in the pathophysiology of RH [4,12,13]. The patient exhibited several clinical characteristics associated with heightened sympathetic drive, including obesity, type 2 diabetes mellitus, obstructive sleep apnoea, and exaggerated exercise-induced hypertensive response [2,12]. Sympathetic activation was considered a likely contributing mechanism [4,6].

A key factor underlying the clinical response in this patient was interruption of the pathophysiological mechanisms associated with sympathetic nervous system (SNS) hyperactivity, which plays a central role in the development and maintenance of hypertension [4,12]. Renal denervation likely disrupted the self-perpetuating cycle in which renal sympathetic activation contributes to sustained elevations in blood pressure [6]. Afferent renal nerve signalling to central autonomic regulatory centres promotes persistent sympathetic activation, while stimulation of efferent renal sympathetic fibres activates β1-adrenergic receptors within the juxtaglomerular apparatus, triggers renin release and activation of the renin–angiotensin–aldosterone system (RAAS) [4,14]. Subsequent sodium retention, water reabsorption, and vasoconstriction contribute to increased intravascular volume and peripheral vascular resistance [1,4,14,15,16].

An exceptionally unique and critical pathophysiological aspect in this case is the patient’s history of a previous nephron-sparing operation. Localised tissue scarring, microcirculatory remodelling and chronic ischaemic changes within the remaining renal parenchyma following partial nephrectomy may generate a persistent state of cellular hypoxia [5]. This structural irritation may act as a potent trigger that continuously hyperactivates the afferent renal nerve pathways, constantly sending signals to the central autonomic centres, intensifying systemic sympathetic drive and rapidly driving the phenotype of resistant hypertension [1,5]. By using radiofrequency energy to selectively ablate these overactive fibres, RDN might alleviate this unique, post-operative neurogenic mechanism [12,16].

The patient’s obesity (BMI 34 kg/m^2^) and type 2 diabetes made him a clinically suitable candidate for renal denervation, as both conditions are associated with chronic sympathetic overactivity [12]. Obesity is linked to increased renal sympathetic outflow, which may be further amplified in the presence of metabolic syndrome and obstructive sleep apnoea, both present in this patient [2,12]. In addition, insulin resistance in diabetes may further enhance sympathetic activity, impair renal sodium handling, and contribute to hypertension [2,12].

Renal denervation likely interrupted both afferent and efferent renal sympathetic signalling, thereby attenuating neurohormonal activation contributing to sustained hypertension [4,6,14]. The evolution of renal denervation technology has substantially improved procedural efficacy and reproducibility [17,18]. Early non-randomised studies such as SYMPLICITY HTN-1 and HTN-2 demonstrated promising blood pressure reductions; however, the neutral findings of SYMPLICITY HTN-3 emphasised the importance of procedural technique, operator experience, medication adherence assessment, and sham-controlled trial design [11,16,19]. Subsequent studies employing second-generation multi-electrode systems, including the Symplicity Spyral™ platform, have demonstrated more consistent reductions in ambulatory and office blood pressure measurements with favourable safety outcomes [17,18].

The use of the multipolar SymplicitySpyral system, equipped with four helically arranged electrodes, allowed controlled delivery of radiofrequency energy while limiting excessive thermal injury to the vessel wall [1,9,14]. This technology facilitates denervation not only within the main renal artery but also in distal branches, where sympathetic nerve fibres are situated closer to the vessel lumen, potentially improving procedural efficacy while maintaining a favourable safety profile [4,16].

Current European Society of Cardiology and European Society of Hypertension guidelines recognise RDN as a potential adjunctive therapy [class IIb recommendation] in selected patients with uncontrolled RH despite optimal medical management and should complement, not replace, guideline-directed pharmacological and lifestyle therapy [7,14,15]. Importantly, careful patient selection remains essential [7,15].

A particularly noteworthy aspect of this case is the patient’s prior nephron-sparing renal surgery [8]. Clinical data regarding RDN in patients with reduced renal mass remain limited, and caution is generally advised in individuals with solitary kidneys or advanced renal dysfunction [7,15]. Nevertheless, no deterioration in renal function was observed during follow-up in this patient, supporting the procedural feasibility of RDN in carefully selected cases following multidisciplinary evaluation [8].

Beyond blood pressure reduction alone, the most clinically meaningful outcome in this case was substantial de-escalation of pharmacotherapy and symptom relief [7]. Reduction in medication burden may improve adherence, reduce adverse effects, and enhance overall quality of life [10]. Importantly, antihypertensive therapy reduction was performed gradually and under close clinical supervision during serial follow-up visits [7].

Despite encouraging outcomes, several limitations must be acknowledged [1,2,14,15]. Responses to RDN remain heterogeneous, and reliable predictors of procedural success have not yet been fully established [1,2,14,15]. Furthermore, although RDN consistently lowers blood pressure, robust evidence demonstrating reductions in major cardiovascular events or mortality remains limited [1,2,9,14,15]. Long-term follow-up is therefore essential, particularly with regard to durability of blood pressure reduction, renal function preservation, and potential late reinnervation phenomena [1,2,4,14,15].

Lifestyle modification remains a cornerstone of RH management [7,14,15]. In addition to dietary sodium restriction, weight reduction, and treatment of obstructive sleep apnoea, growing evidence supports structured resistance training and patient self-management programmes as valuable adjunctive interventions for blood pressure control [13,20,21,22].

## 4. Study Limitations

The main limitation of this study is its nature, which stems from the fact that it is a single-case analysis (case report). Although a significant reduction in blood pressure was observed in the patient during clinic measurements and 24 h ambulatory blood pressure monitoring (ABPM), and the number of antihypertensive medicines taken was successfully reduced, these results relate to only one specific individual. Consequently, the conclusions drawn cannot be directly generalised to the entire population of patients with resistant hypertension. Furthermore, although this case demonstrates the potential role of RDN in a patient with a specific surgical history (previous parenchymal-sparing renal surgery), individual anatomical variations and vascular reactivity may influence the final therapeutic outcome. Therefore, although the results obtained are very encouraging, larger prospective cohort studies and registry analyses are needed to confirm the safety and long-term efficacy of renal denervation in this specific patient group.

## 5. Conclusions

This case demonstrates that catheter-based renal denervation may represent an effective adjunctive therapeutic strategy in carefully selected patients with true RH and marked sympathetic overactivity. In the present patient, RDN achieved substantial and sustained improvement in ambulatory blood pressure control while enabling a major reduction in the antihypertensive medication burden through the complete withdrawal of 4 distinct classes of antihypertensive medications (centrally acting agents, loop diuretics, mineralocorticoid receptor antagonists, and long-acting nitrates) alongside a substantial dose reduction in alpha-1 blockers. Beyond haemodynamic benefit alone, simplification of pharmacotherapy may improve adherence and quality of life, with the potential to support long-term cardiovascular risk management. It should be clearly noted, however, that the observations and conclusions presented here are based on a single-case report. For this reason, the results obtained cannot be directly generalised to the entire population of patients with resistant hypertension, and further prospective studies involving larger groups of patients are required to confirm their efficacy.

## Data Availability

The original contributions presented in this manuscript are included in the references section. Further inquiries can be directed to the corresponding author.
